# HMGB1 lactylation drives neutrophil extracellular trap formation in lactate-induced acute kidney injury

**DOI:** 10.3389/fimmu.2024.1475543

**Published:** 2025-01-09

**Authors:** Li Zhu, Qiang Zheng, Xiaodong Liu, Hao Ding, Mengqing Ma, Jiaxin Bao, Yawen Cai, Changchun Cao

**Affiliations:** ^1^ Department of Nephrology, Sir Run Run Hospital, Nanjing Medical University, Nanjing, Jiangsu, China; ^2^ Department of Nephrology, Affiliated People’s Hospital of Jiangsu University, Zhenjiang, Jiangsu, China; ^3^ The Second People’s Hospital of Lianyungang, Affiliated to Kangda College of Nanjing Medical University, Lianyungang, Jiangsu, China; ^4^ Department of Respiratory Disease, Affiliated People’s Hospital of Jiangsu University, Zhenjiang, Jiangsu, China

**Keywords:** lactate, HMGB1 lactylation, neutrophil extracellular traps (NETs), acute kidney injury, LPS

## Abstract

**Rationale:**

Acute kidney injury (AKI) is a clinical syndrome associated with a multitude of conditions. Although renal replacement therapy (RRT) remains the cornerstone of treatment for advanced AKI, its implementation can potentially pose risks and may not be readily accessible across all healthcare settings and regions. Elevated lactate levels are implicated in sepsis-induced AKI; however, it remains unclear whether increased lactate directly induces AKI or elucidates the underlying mechanisms.

**Methods:**

For human, the measurement of lactate in arterial blood gas is performed using the direct determination of L-lactate through an electrode oxidation method by a blood gas analyzer. For mice, enzyme-linked immunosorbent assay (ELISA) kits were employed to quantify the concentrations of lactate and AKI biomarkers in blood and cell supernatant. The mouse model of AKI was performed with a single intraperitoneal (*i.p.*) administration of lactate (30 mg/kg) and low-dose LPS (2 mg/kg) for 24 h. Proteomic analysis was conducted to identify lactylated proteins in kidney tissues. Techniques such as, immunoprecipitation, western blotting and immunofluorescence were used to evaluate the levels of HMGB1 lactylation, neutrophil extracellular traps (NETs)and to assess related molecular signaling pathways.

**Main results:**

Our findings indicate that lactate serves as an independent predictor of AKI in patients with acute decompensated heart failure (ADHF). We observed that co-administration of lactate with low-dose lipopolysaccharide (LPS) resulted in lactate overproduction, which subsequently elevated serum levels of creatinine (Cre) and blood urea nitrogen (BUN). Furthermore, the combined application of lactate and low-dose LPS was shown to provoke HMGB1 lactylation within renal tissues. Notably, pretreatment with HMGB1 small interfering RNA (siRNA) effectively diminished lactate-mediated HMGB1 lactylation and alleviated the severity of AKI. Additionally, lactate accumulation was found to enhance the expression levels of NETs in the bloodstream, with circulating NETs levels positively correlating with HMGB1 lactylation. Importantly, pre-administration of HMGB1 inhibitors (glycyrrhizin) or lactate dehydrogenase A (LDH-A) inhibitors (oxamate) reversed the upregulation of NETs induced by lactate and low-dose LPS in both the blood and polymorphonuclear neutrophils (PMNs) cell supernatant, thereby ameliorating AKI associated with lactate accumulation.

**Conclusions:**

These findings illuminate the role of lactate-mediated HMGB1 lactylation in inducing AKI in mice through the activation of the HMGB1-NETs signaling pathway.

## 1 Introduction

Acute kidney injury (AKI) is a clinical syndrome characterized by a rapid decline in renal function over a short duration, typically manifesting as a decreased glomerular filtration rate, accumulation of nitrogen metabolic end products such as creatinine and urea, and disturbances in electrolytes and acid-base balance. The incidence of AKI among hospitalized patients is approximately 10% to 15% (1), and it exceeds 50% in intensive care settings (2). While conventionally perceived as a singular condition, AKI is actually a composite entity intertwined with various syndromes including hepatorenal syndrome (3), cardiorenal syndrome (4), nephrotoxic syndrome (5), each exhibiting unique pathophysiologies that vary according to the specific syndrome involved (6). In animal models of sepsis, although the histopathological changes in renal tissue within the initial 48 h may not be prominent, there is a significant increase in renal blood flow, coupled with markedly elevated levels of serum Cre and BUN (7, 8). Moreover, factors such as low cardiac output, hypoperfusion, and hypoxemia occurring after major surgeries (e.g., cardiac procedures) may also precipitate AKI (9). As a result, AKI serves not only as an independent predictor of morbidity and mortality but also as a complication associated with particularly adverse outcomes when it occurs concurrently with other diseases. Nevertheless, the underlying mechanisms contributing to this multifactorial nature of AKI remain inadequately understood.

Lactate, first isolated from milk in 1780, was historically considered merely a metabolic waste product of glycolysis (10). Recent studies, however, highlight lactate’s role as an energy source and its production even under aerobic conditions (11). Lactate contributes significantly to energy production in the brain, especially in the absence of glucose (12) and acts as a redox buffer vital for maintaining redox homeostasis (13–15). Nonetheless, excessive lactate accumulation can result in various pathological conditions, such as hyperlactatemia (16), lactic acidosis (17), and inflammatory diseases (18). Research has shown that lactate can upregulate Toll-like receptor 4 (TLR4) signaling, activating the NF-κB pathway and promoting the expression of inflammatory genes like interleukin-6 (IL-6) and interleukin-1β (IL-1β) in macrophages (19). Lactate can also enhance its own transporter, SLC5A12, facilitating entry into CD4^+^ T lymphocytes and promoting IL-17 production through the PKM2/STAT3 pathway (20). Furthermore, inhibition of LDH-A has been shown to considerably reduce the inflammatory response and mitigate the depletion of CD8^+^ T lymphocytes in a rheumatoid arthritis mouse model (21).

The 2019 Nobel Prize in Physiology or Medicine underscored the significance of hypoxia in disease pathogenesis, including tumors (22), infections (23), ischemia (24), and inflammation (25, 26). Hypoxia-inducible factor 1α (HIF-1α) has emerged as a pivotal transcription factor that orchestrates cellular responses to hypoxia, exhibiting stabilized expression and nuclear translocation to regulate downstream gene expression under low oxygen conditions (27, 28). Hypoxia-driven glycolysis fosters lactate production (29), which can modify histone lysine residues through a process called lactylation, influencing gene transcription (30), and implicated in various pathologies such as tumors (31), sepsis (32), and immune-related disorders (33). Additionally, both lactate accumulation and lactylation have been identified as contributing factors in sepsis-related AKI. Xu et al. demonstrated that lactate can activate the PD-1/PD-L1 signaling pathway, inducing immune suppression and promoting apoptosis of lymphocytes, while pretreatment with a lactate receptor inhibitor or blockade of PD-1/PD-L1 signaling can alleviate sepsis-induced AKI (34). Elevated lactate levels also facilitate Fis1 lactylation, exacerbating AKI, while reducing lactate correlates with improved outcomes (35). Furthermore, lactate levels are positively correlated with HMGB1 concentrations in the blood of sepsis patients (36). Lactate can induce HMGB1 lactylation through a p300/CBP-dependent mechanism, thereby exacerbating the progression of sepsis (32). However, the direct relationship between elevated lactate and the causation of AKI, along with the underlying mechanism, remains unclear. In this study, we first conducted a retrospective analysis of clinical data to assess the potential of lactate as an independent risk factor for AKI. Subsequently, we investigated whether lactate accumulation can directly induce AKI in mice and explored the underlying mechanisms.

## 2 Materials and methods

### 2.1 Subjects

The clinical electronic medical records of patients from the cardiovascular, neurology, and intensive care units of Zhenjiang First People’s Hospital were collected and analyzed for this study from March 2018 to March 2022. An early warning model was developed based on an analysis of risk factors and clinical characteristics. Exclusion criteria were established, excluding patients with hospital stays, those undergoing dialysis treatment while hospitalized (eGFR < 30 ml/min), and individuals with other conditions such as pregnancy, tumors, psychosis, non-diabetic cerebral hemorrhage, cardiac surgery, urinary tract infections, or those who had been exposed to nephrotoxic agents (e.g., contrast media, aminoglycosides, vancomycin). Patients were categorized into two groups based on the occurrence of AKI: the AKI group and the non-AKI group. A further analysis of patient characteristics was performed using univariate analysis, supplemented by chi-square tests, independent sample t-tests, and non-parametric tests. Significant variables identified during the univariate analysis were incorporated into a multivariate logistic regression model. The predictive accuracy of the model was assessed using the area under the receiver operating characteristic (ROC) curve (AUC), with the model exhibiting the highest AUC value selected as the final model. The final selected logistic regression model was visualized through nomograms, allowing each patient to be scored accordingly, facilitating the estimation of event probabilities based on the total score. Informed oral consent was obtained from all patients, and the study protocol received approval from the ethics committee of Zhenjiang First People’s Hospital (V3.0/20180113).

### 2.2 Chemicals and reagents

Glycyrrhizin, L-Sodium lactate, lipopolysaccharide (LPS)and sodium oxamate were purchased from Sigma-Aldrich (Merck KGaA). RS 09 was obtained from MedChemExpress (MCE). ELISA kits were sourced from R&D Systems (Minn, USA). Primary antibodies against neutrophil elastase (NE) and citrullinated histone H3 (citH3) were acquired from Abcam (Cambridge, MA, USA). Antibodies for tissue factor (TF), hypoxia-inducible factor 1α (HIF-1α), high mobility group box 1 (HMGB1), and β-actin were obtained from Cell Signaling Technology (Beverly, MA, USA). Additionally, antibodies for pan-lactylation (pan-Kla) were sourced from PTM BIO Co. LTD. Fetal bovine serum (FBS) was purchased from Gibco (Thermo Fisher Scientific), while other cell culture media and supplements were obtained from Nanjing KeyGen Biotech Co., Ltd. (KenGEN, China). All other reagents were procured from Sigma-Aldrich (Merck KGaA).

### 2.3 Animals and treatment

Elderly (18-20 months old) male CD-1 mice weighted 18-22 g were purchased from the Experimental Animal Center at Nanjing Medical University, Nanjing, China. The mice were housed in groups of five to six in pathogen-free environments with soft bedding, maintained at a controlled temperature of 22 ± 2°C, and subjected to a 12-hour light/dark cycle, with lights turning on at 8:00 a.m. All experiments adhered to protocols approved by the institutional review board. Animal experiments received ethical approval from the Animal Care and Ethics Committee of Nanjing Medical University, under the reference number IACUC2202017.

To develop the AKI model, mice were randomized into two groups (n = 10 each): the Control group and the Lactate group or the Lactate + low-dose LPS group. Three different methodologies were employed to induce AKI in elderly mice. The first method involved a single intravenous (*i.v.*) injection of 50 mg/kg lactate. The second method involved three *i.v.* injections of 50 mg/kg lactate administered every 12 h. Lastly, an intraperitoneal (*i.p.*) combination treatment was performed with a single administration of lactate (30 mg/kg) and low-dose LPS (2 mg/kg).

To assess the impact of HMGB1 inhibitor (glycyrrhizin) and LDH-A inhibitor (oxamate) on kidney tissue damage in mice, animals received *i.p*. administration of glycyrrhizin at a dose of 30 mg/kg or oxamate at a dose of 750 mg/kg for 2 h. ​This was followed by a combined treatment with lactate and low-dose LPS for 24 h.

### 2.4 Cell culture

HK2 cells were purchased from the American Type Culture Collection and were maintained in Dulbecco’s modified Eagle’s medium (DMEM) from KenGEN Bio TECH, China. This medium was supplemented with 10% (v/v) FBS, along with penicillin (100 U/ml) and streptomycin (100 U/ml). We used HK2 cells from passages 5 to 10 to ensure that the cells were in a healthy proliferative state. 1×10^6^ cells were plated per well in 6-well plates for about 48 h cultured in a humidified incubator at 37°C with 5% CO_2_ until they reached approximately 70-80% confluency.

Murine polymorphonuclear neutrophils (PMNs) were isolated from the bone marrow of male C57BL/6 mice, following strict institutional animal care guidelines. After euthanization, the femurs and tibias were excised, and their epiphyses removed to facilitate bone marrow extraction. Sterile phosphate-buffered saline (PBS) was then used to flush the bone marrow from the bones, which was subsequently collected and filtered through a 70 µm cell strainer to obtain a single-cell suspension. The purity of PMNs was achieved through density gradient centrifugation using EasySep kits from Stem Cell Technologies (37), where the cell suspension was treated with specific antibodies targeting non-PMN cells, followed by the addition of magnetic particles for separation. After washing the isolated PMNs with sterile PBS to eliminate residual antibodies and particles, they were resuspended in RPMI 1640 culture medium supplemented with 10% FBS and penicillin (100 U/ml) and streptomycin (100 U/ml) and maintained in a humidified incubator with 5% CO_2_ at 37°C.

### 2.5 Enzyme linked immunosorbent assay measurement

In accordance with the manufacturer’s guidelines, the Amplite™ Fluorimetric Acidic Sphingomyelinase Assay Kit (R&D Systems, Minn, USA) was employed to quantify levels of NE, myeloperoxidase (MPO), citH3, Cre, BUN, lactate, and HMGB1 in both blood and kidney tissue samples. Samples were collected at various time points (0, 12, 24, 36, and 48 h), with kidney tissues harvested 48 h after the final administration of lactate or the lactate + low-dose LPS treatment. After homogenization and centrifugation at 1500 rpm for 5 min at 4°C, the supernatants were collected for protein analysis. Quantification of protein concentration was performed using a BCA protein assay kit, with optical density (OD) values recorded at 405 nm.

### 2.6 Hematoxylin and eosin

Mice were anesthetized with 1% sodium amobarbital, and the kidney tissue was promptly collected for histological analysis. The samples underwent dehydration in a sequence of graded ethanol solutions prior to being embedded in paraffin after fixation in 10% formalin for 24 h. Subsequently, HE staining was conducted on sections cut at a thickness of 4 micrometers using a microtome.

### 2.7 Small interfering RNA

The HMGB1 siRNA (5′-CAGGAGGAATACTGAACATTT-3′) and control siRNA (5′-TTCTCCGAACGTGTCACGTTT-3′) were packaged using lentivirus and obtained from Gene Pharma Co. (Shanghai, China). A concentration of 3.3 nmol of siRNA was dissolved in 330 μl of RNase-free water. ​For the administration, 2 nmol of siRNA were injected intraperitoneally for every 20 grams of mouse body weight, with the control siRNA serving as a negative control. For siRNA transfection, cells were cultured in six-well plates until confluence reached 60-80%. The Lipofectamine 2000 reagent (Invitrogen, USA) was coated with siRNA according to the manufacturer’s instructions. After 6 h, the transfection medium was replaced with a culture medium containing 10% FBS, followed by incubation at 37°C in a 5% CO_2_ atmosphere. For animal experiments, siRNA was administered intraperitoneally into mice 48 h prior to treatment with lactate + low-dose LPS.

### 2.8 Immunoprecipitation

Immunoprecipitation was conducted as previously described (38). In brief, approximately 200 μg of total cellular proteins were incubated overnight at 4°C with antibodies, including pan-Kla and HMGB1. Following this, 20 μL of protein A/G-agarose beads (Santa Cruz Biotechnology) was added. The precipitates were washed four times with lysis buffer and boiled in SDS sample buffer. The supernatant was subsequently subjected to immunoblotting using the appropriate antibodies.

### 2.9 Western blotting

Kidney samples were lysed using RIPA buffer, and protein concentrations were determined using a BCA assay. Equal amounts of protein were loaded onto a gel, separated by SDS-PAGE, and subsequently transferred to polyvinylidene fluoride membranes. The membranes were blocked with 5% bovine serum albumin for 2 h at room temperature, followed by overnight incubation with primary antibodies at 4°C. HRP-coupled secondary antibodies were then applied. The primary antibodies used in this study targeted NE, citH3, TF, HIF-1α, HMGB1, and β-actin. The blots were probed with an antibody against β-actin as a loading control. Detection was achieved using enhanced chemiluminescence reagents, and data were collected using a Molecular Imager (Gel Doc™ XR, 170-8170). Analysis was conducted with Quantity One-4.6.5 software (Bio-Rad Laboratories, Berkeley, CA, USA).

### 2.10 Immunofluorescence assay

Confocal microscopy was employed to visualize and localize NETs within kidney tissue. Following administration of LPS and lactate into mice, kidney samples were collected 48 h later. Kidney sections were fixed in 4% paraformaldehyde, permeabilized in 0.5% Triton X-100, and then incubated with a NE antibody (diluted in PBS at 1:200) overnight at 4°C after being blocked with 10% donkey serum in PBS for 2 h. After exposure to FITC-conjugated anti-rabbit IgG (1:300) at room temperature for 2 h, coverslips were washed three times with PBS. Lastly, coverslips were stained with DAPI for 5 min. A Carl Zeiss LSM900 confocal system was utilized for confocal microscopy analysis.

### 2.11 LC-ESI-MS/MS analysis

Analyses were performed using a Thermo Scientific Q ExactiveTM mass spectrometer (San Jose, USA) coupled with a nanoACQUITY UPLC Waters system (Milford, MA, USA). For chromatographic runs, 2 μL of sample were loaded onto a PST C18 nanoACQUITY Trap column (180 μm × 20 mm) with a flow rate set to 15 μL/min of 0.1% (vol/vol) trifluoroacetic acid for 3 min. The analytical separation of peptides was conducted on a nanoACQUITY UPLC HSS C18 Column (1.8 μm, 75 μm × 150 mm), employing a linear gradient of 90 min from 1% ACN/0.1% formic acid to 60% ACN/0.1% formic acid at a flow rate of 0.5 μL/min. Spectra were acquired over the m/z range of 300–2000 at a resolution of 70,000 (m/z 200). Data-dependent acquisition selected the top 10 most abundant precursor ions for tandem mass spectrometry using high-energy collision dissociation (HCD) fragmentation with an isolation width of 1.2 Da, collision energy of 30, and a resolution of 35,000. Each biological replicate was analyzed once.

### 2.12 Statistical analyses

All statistical analyses were performed using the GraphPad Prism 9 software (GraphPad Software, San Diego, CA, USA). Kaplan-Meier survival analysis was executed utilizing the log-rank (Mantel-Cox) test. The data were statistically evaluated using one-way or two-way analysis of variance (ANOVA) followed by Bonferroni *post-hoc* tests. ​Results are represented as mean ± SEM of at least three independent experiments, and a significance threshold was established at *P* < 0.05.

## 3 Results

### 3.1 Lactate is an independent predictor of AKI in patients with ADHF

A total of 194 patients diagnosed with ADHF were included in this study, of which 85 patients, representing 43.8%, were diagnosed with AKI. This cohort included 45 patients classified with AKI stage 1, 10 with AKI stage 2, and 8 with AKI stage 3. Patient characteristics and the prevalence of Cardiorenal Syndrome are detailed in **Table 1**. The results of the univariate analysis are presented in **Table 2**, while adjustments for multiple variables are shown in **Table 3**. Notably, lactate levels, neutrophil-to-lymphocyte ratios, pro-BNP, and hemoglobin were identified as independent predictors of AKI in patients with ADHF (*P* < 0.05).

**Table 1:** Baseline table, variable distribution of ADHF patients with or without AKI.

Characteristic		Total patients (N=194)	Non-AKI group (N=109)	AKI group (N=85)	P
Hospital stays		9.5 ± 3.5	9 ± 3	9.25±4.25	0.811
Gender	Male	106	58	48	0.38
	Female	88	51	37	0.176
Age		77.5	75.0	79.0	0.176
Smoking		58	34	24	0.388
Myocardial Infarction (MI)		19	9	10	0.282
Cardiopathy		193	108	85	0.282
NYHA class	III	87	59	28	0.002
	IV	107	50	57	
Hypertension		154	84	70	0.235
COPD		20	11	9	0.546
CKD		74	32	42	0.003
Cerebrovascular		30	16	14	0.441
Diabetes		70	44	26	0.104
Infection		80	40	40	0.096
Disturbance of consciousness		12	2	10	0.005
Body temperature		36.8	36.8	36.7	0.333
Sphygmus		80	82	78	0.432
Breathing rate		18	18	18	0.142
Systolic blood pressure		136.56±26.239	137.41±25.466	135.46±27.312	0.405
Diastolic blood pressure		76.5	77	76	0.508
hsCRP > 8 mg/L		7.6	5.02	16.23	< 0.001
Lactate ≥ 2.2 mmol/L		1.9	1.6	2.6	< 0.001
P O_2_ ≤ 90%		87.75	90	80.5	0.203
Neutrophil/lymphocyte ≥11		4.6	3.5	8.9	< 0.001
PRO		43	16	27	< 0.001
BLD		44	26	18	0.462
cTnI > 0.1 ng/ml		0.02	0.02	0.04	0.029
Pro-BNP ≥ 450 pg/ml		3931.55	1347.6	10435.17	< 0.001
D2 polymers (0.1-0.55mg/l)		0.94	0.82	1.29	< 0.001
ALB		33.7	34.2	33.55	0.202
LDL		1.77	1.68	1.82	0.076
TC		3.67	3.58	3.75	0.483
PCT		0.3	0.14	0.54	< 0.001
FPG		6.07	5.93	6.24	0.16
BUN		8.75	7.9	10.4	0.025
CRE		103	90.2	116.69	< 0.001
UA		458.55±175.8	447.74±174.9	472.57±117.02	0.743
GFR ml/min		61.39±31.74	68.21±34.49	52.64±25.45	0.004
Serum kalium		3.99±0.65	4.02±0.69	3.95±0.61	0.27
Serum natrium		139.2±4.85	138.98±4.39	139.49±5.39	0.508
EF < 50 %		46.09±14.93	48.78±14.87	42.66±14.37	0.608
NK1 antagonist		80	50	30	0.146
Spironolactone		113	70	43	0.117
Loop diuretics		161	92	69	0.015
CCB		79	45	34	0.044
ACEI		41	28	13	0.538
ARB		29	20	9	0.544
Antidiabetic		64	36	28	0.033
Vasoconstrictor		27	7	20	< 0.001
Vasodilators		69	43	26	0.275
Digoxin		96	47	49	0.021
Statins		105	65	40	0.268
Anticoagulation		160	93	67	0.376
CRRT		20	1	19	< 0.001
Mechanical ventilation		45	14	31	< 0.001

**Table 2:** Univariate analysis of AKI in patients with ADHF.

General condition	OR	95%CI	P
NYHA class (III VS IV)	2.402	(1.333,4.328)	0.004
CKD (with or without)	2.35	(1.3,4.249)	0.005
Disturbance of consciousness (with or without)	7.133	(1.519,33.495)	0.013
Breathing rate	1.113	(1.027,1.206)	0.009
Hb	0.981	(0.968,0.993)	0.002
hsCRP classification (> 8 mg/L VS ≤8 mg/L)	0.292	(0.16,0.533)	<0.001
Lactate (≥ 2.2 mmol/L VS < 2.2 mmol/L)	4.879	(2.415,9.855)	<0.001
Neutrophil/lymphocyte ≥11 (≥ 11 VS < 11)	15.455	(6.11,39.093)	<0.001
PRO (Positive VS negative)	4.746	(2.046,11.008)	<0.001
cTnI (> 0.1ng/ml VS ≤ 0.1ng/ml)	2.12	(1.004,4.479)	0.049
Pro-BNP (≥ 450 pg/ml VS <450 pg/ml)	4.236	(1.479,12.136)	0.007
D2 polymers (0.1-0.55 mg/l VS other)	0.441	(0.226,0.858)	0.016
ALB (<30 g/l VS ≥30g/l)	2.102	(1.064,4.151)	0.032
CRE	1.009	(1.004,1.014)	0.001
GFR (< 50 %VS ≥50%)	0.983	(0.973,0.993)	0.001
EF	2.894	(1.537,5.447)	0.001
Loop diuretics (with or without)	8.25	(1.04,65.431)	0.046
Antidiabetic (with or without)	2.274	(1.021,5.061)	0.044
Vasoconstrictor (with or without)	5.952	(2.214,16)	<0.001
Digoxin (with or without)	2.04	(1.073,3.877)	0.03
CRRT (with or without)	39.966	(5.099,313.224)	<0.001
Mechanical ventilation (with or without)	4.658	(2.155,10.065)	<0.001

**Table 3:** Multi-factor logistic regression of AKI in ADHF patients.

	Coefficient	OR	95% CI	P
Intercept	0.32	1.38	(0.06, 25.77)	0.832
Lactate (≥ 2.2 mmol/L VS < 2.2 mmol/L)	1.40	4.06	(1.50, 11.69)	0.007
Neutrophil/lymphocyte ≥11 (≥ 11 VS < 11)	2.92	18.51	(5.39, 88.36)	< 0.001
Pro-BNP (≥ 450 pg/ml VS <450 pg/ml)	2.54	12.74	(2.02, 126.46)	0.018
Hb	-0.04	0.96	(0.94, 0.99)	0.001

**Figure 1:**
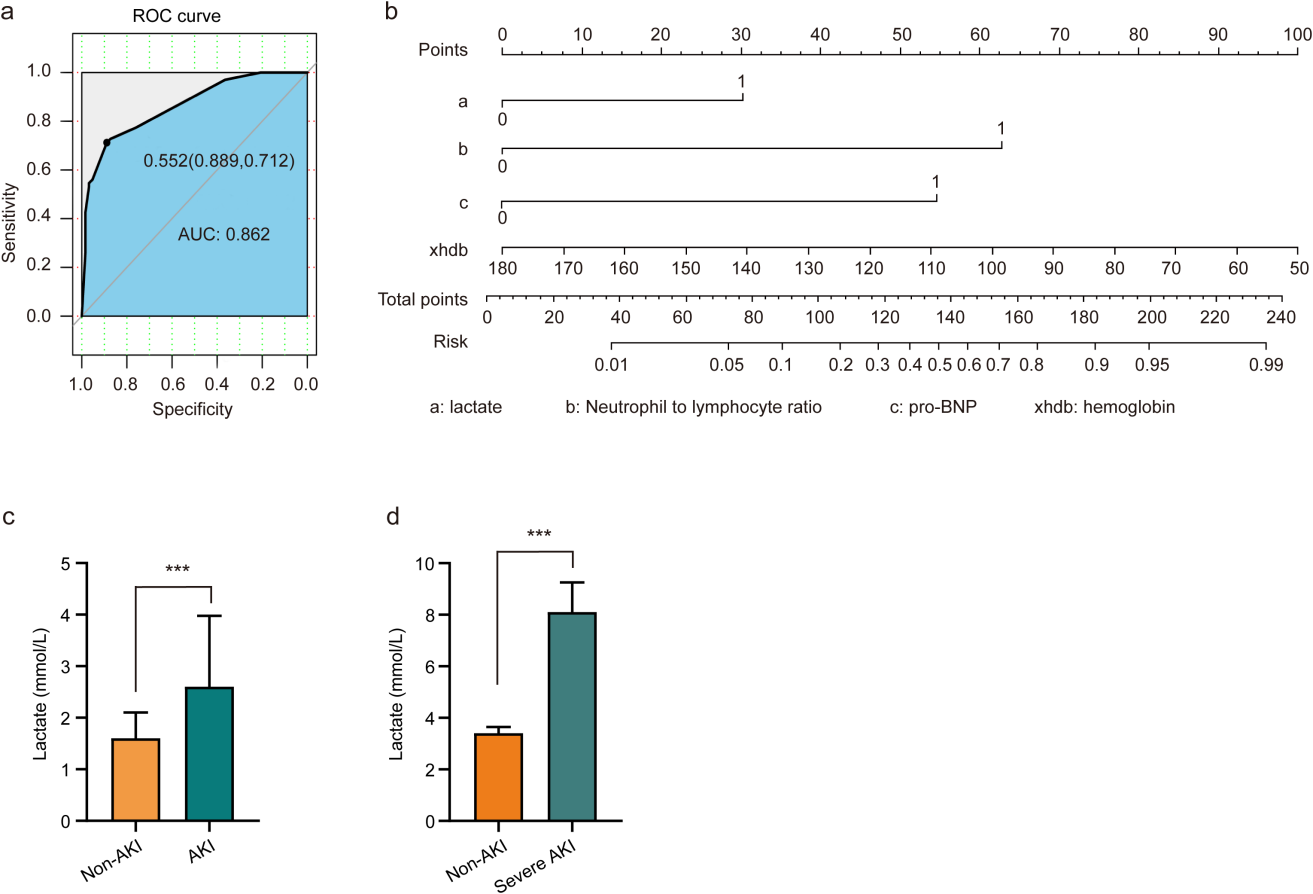
Lactate serves as an independent predictor of AKI in patients with ADHF. **(A)** Risk factors of AKI were analyzed by multivariate logistic regression, and the ROC curve was constructed. **(B)** Nomograms employed as prognostic tools for assessing AKI in ADHF patients. **(C)** Patients diagnosed with AKI exhibited elevated lactate levels compared to non-AKI patients. **(D)** Patients with severe AKI have higher lactate levels than non-AKI patients. Significant difference was revealed following one-way ANOVA (^***^ *P* < 0.001 vs. non-AKI group; Bonferroni *post hoc* tests).

To assess the risk factors for AKI, multivariate logistic regression was performed, and a ROC curve was constructed. As depicted in **Figure 1A**, the AUC was 0.862, indicating a high predictive value and reflecting the model’s robust ability to forecast the occurrence of cardiorenal syndrome. The risk of cardiorenal syndrome heightened in accordance with escalating abnormal biomarker levels. Furthermore, the nomogram revealed that lactate, neutrophils/lymphocytes, pro-BNP, and hemoglobin collectively predicted the risk coefficient for AKI. The total score of 209 indicated a greater than 95% probability of AKI occurrence, demonstrating strong predictive capabilities, as illustrated in **Figure 1B**. Evidently, patients with AKI exhibited significantly higher lactate levels compared to those without AKI (**Figures 1C, D**). Overall, these findings underscore that lactate is an independent predictor of AKI in patients with ADHF.

### 3.2 Lactate accumulation induced AKI in mice

**Figure 2:**
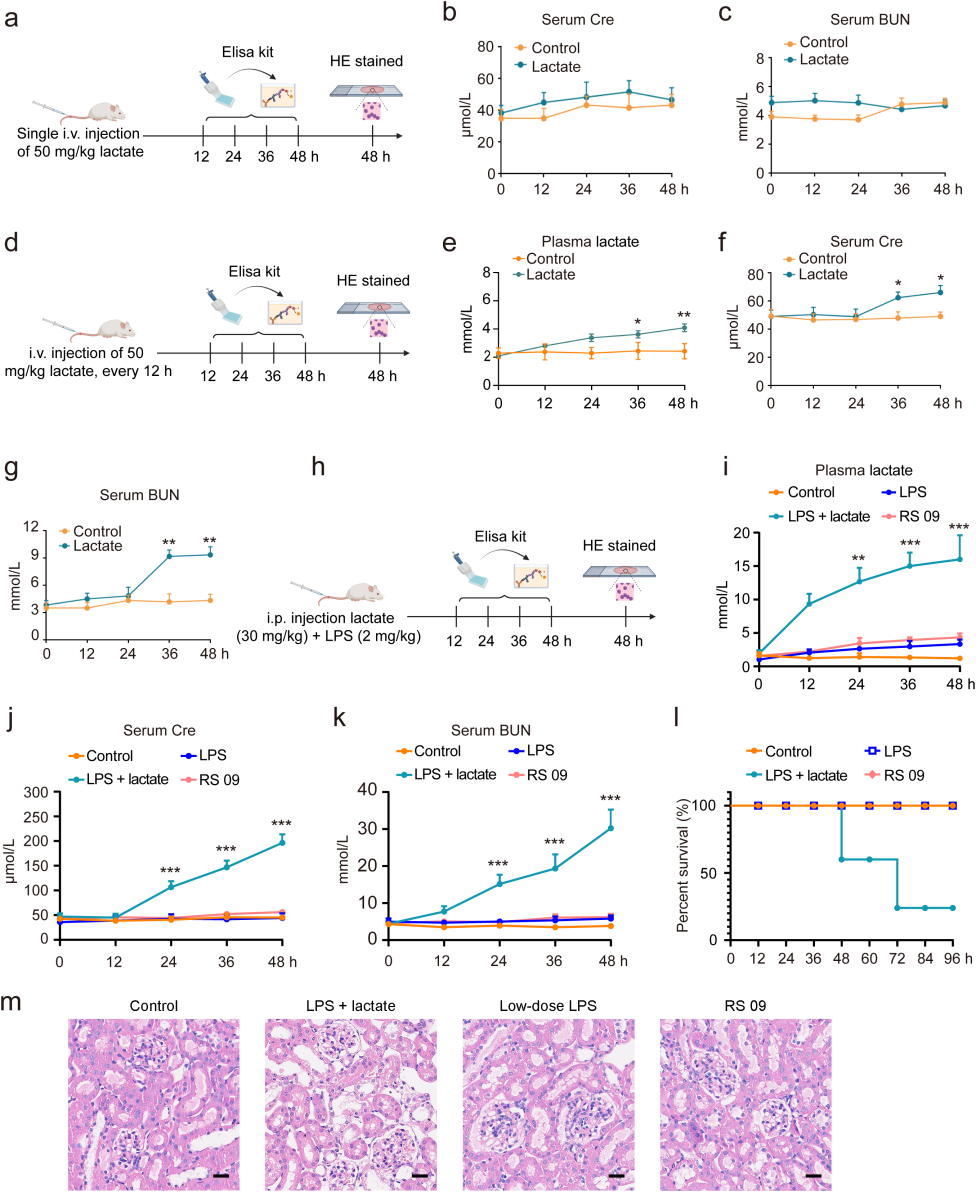
Lactate accumulation is implicated in the induction of AKI in mice. **(A)** A schematic representation outlines the experimental protocol for AKI development in mice. *I.v.* injection of 50 mg/kg lactate to mice for once, followed by serum collection at intervals of 0, 12, 24, 36, and 48 h to quantify serum Cre and BUN levels. ELISA kits were utilized to assess Cre **(B)** and BUN **(C)** serum levels. **(D)** The experimental procedure for AKI development involved the administration of 50 mg/kg lactate intravenously every 12 h, resulting in a slight elevation of lactate **(E)**, Cre **(F)**, and BUN **(G)** levels in serum at 36 h. **(H)** A schematic depiction indicates that the combined application of lactate (30 mg/kg) and low-dose LPS (2 mg/kg) significantly increased lactate **(I)**, Cre **(J)**, and BUN **(K)** levels in the serum in mice. **(L)** Kaplan-Meier survival analysis revealed the survival rate of mice subjected to lactate and low-dose LPS treatment. Log-rank (Mantel-Cox) testing was employed to determine significance (n = 10 for each group). **(M)** Hematoxylin- and Eosin (HE) stained sections of the kidney tissue of from mice are shown. Scale bar: 100 μm. Significant difference was revealed following one-way ANOVA (^*^ *P* < 0.05, ^**^ *P* < 0.01, ^***^ *P* < 0.001 vs. control group; Bonferroni *post hoc* tests).

In this investigation, various methodologies were employed to determine whether lactate accumulation induces AKI in mice. A single *i.v.* injection of 50 mg/kg lactate did not elevate serum Cre or BUN levels within 48 h (**Figures 2A–C**). However, with repeated *i.v.* injections of 50 mg/kg lactate every 12 h, there was a moderate increase in lactate levels observed at 36 h, alongside only slight elevations in serum Cre and BUN levels (**Figures 2D–G**). Prior research indicated a significant increase in blood lactate concentrations during states of infection or enhanced glycolysis (39). Consequently, we combined a lactate dose of 30 mg/kg with low-dose LPS (2 mg/kg) to establish a mouse model of AKI. The results demonstrated that the combination of lactate and low-dose LPS substantially augmented the levels of lactate, Cre, and BUN in the bloodstream (**Figures 2H–K**). Further validation focused on whether the combination of lactate and low-dose LPS exacerbates AKI by inducing elevated lactate levels in the blood. Notably, neither the LPS mimic peptide (RS 09, 2 mg/kg), a TLR4 agonist, nor low-dose LPS (2 mg/kg) alone resulted in increased serum Cre and BUN levels (**Figures 2J, K**). Additionally, neither the low-dose LPS group nor the RS 09 group showed mouse mortality within 48 h compared to the lactate + low-dose LPS group (**Figure 2L**). Histological examination via HE staining corroborated that the combination of lactate and low-dose LPS induces vacuolar degeneration of renal tubular epithelial cells and neutrophil infiltration (**Figure 2M**). Collectively, these findings indicate that lactate accumulation can instigate AKI in mice.

### 3.3 Lactate-induced AKI through HMGB1 lactylation in kidney tissues

**Figure 3:**
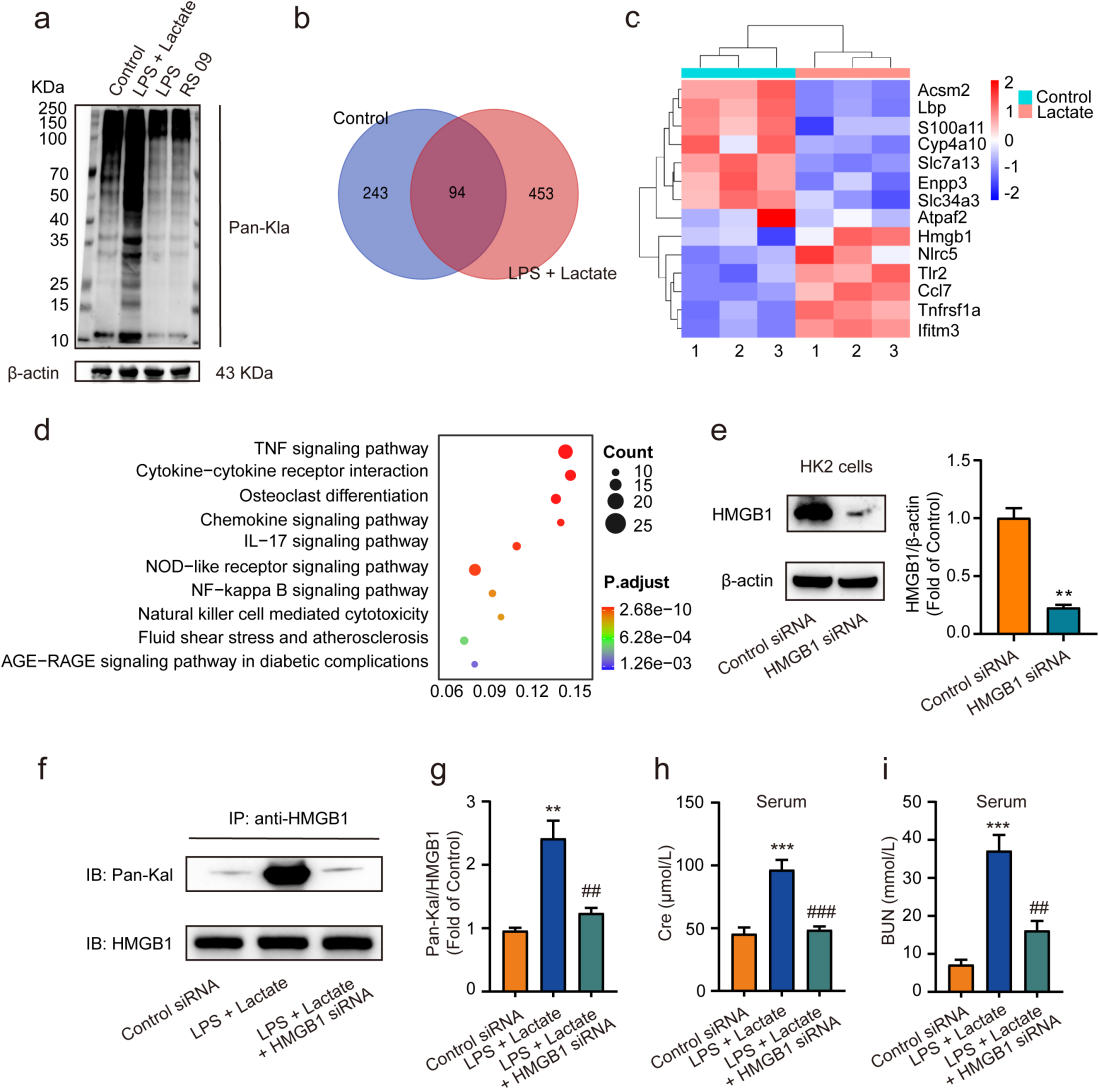
Lactate-induced AKI mediated by HMGB1 lactylation in kidney tissues. **(A)** The levels of protein lactylation were assessed by western blotting following treatment with lactate and low-dose LPS for 48 h in mouse kidney tissues. **(B)** A Venn diagram illustrates the number of genes with changes in control group compared to the LPS + lactate-treated group. **(C)** A heatmap visualization displays the most significantly altered genes in kidney tissues after LPS + lactate treatment. **(D)** The KEGG enrichment scatter plot depicts the pathways of lactylation proteins identified. **(E)** HMGB1 siRNA transfected into HK2 cells significantly reduced HMGB1 expression, with control siRNA serving as a loading control. **(F, G)** The effects of HMGB1 siRNA treatment on lactate and low-dose LPS induced HMGB1 lactylation in mice are shown. **(H, I)** The effects of HMGB1 siRNA treatment on lactate and low-dose LPS induced the upregulation of Cre and BUN in serum are illustrated. Significant difference were revealed following one-way ANOVA (^**^ *P* < 0.01, ^***^ *P* < 0.001 vs. control siRNA group; ^##^ *P* < 0.01, ^###^ *P* < 0.001 vs. lactate + low-dose LPS group Bonferroni *post hoc* tests).

Recent studies have revealed that the accumulation of lactate contributes to various diseases through lactylation modifications (40, 41). In our study, we observed that the combined application of lactate and low-dose LPS significantly elevated the lactylation levels of several proteins in kidney tissues, in contrast to the effects of low-dose LPS alone or the LPS mimic peptide (RS 09) group (**Figure 3A**). Employing proteomic analysis, we identified numerous genes that were upregulated, including HMGB1 (**Figures 3B, C**). Furthermore, pathway enrichment analysis via KEGG indicated that these upregulated lactylated proteins are closely associated with inflammatory responses, as well as NF-κB and advanced glycation end-product-specific receptor (RAGE) signaling pathways (**Figure 3D**). Further validated by HMGB1 siRNA, we found that treatment with HMGB1 siRNA (10 nM) for 48 h significantly reduced HMGB1 expression levels in HK2 cells *in vitro* compared to the control siRNA group (**Figure 3E**). Additionally, pre-administration of HMGB1 siRNA into mice 48 h prior to AKI induction using lactate and low-dose LPS was performed. Western blot analysis was conducted to evaluate HMGB1 lactylation levels in kidney tissues. The results demonstrated that HMGB1 siRNA significantly diminished HMGB1 lactylation levels in the kidney tissues of AKI model mice (**Figures 3F, G**), as well as decreased serum levels of Cre and BUN (**Figures 3H, I**).

### 3.4 HMGB1 lactylation induces the release of NETs

**Figure 4:**
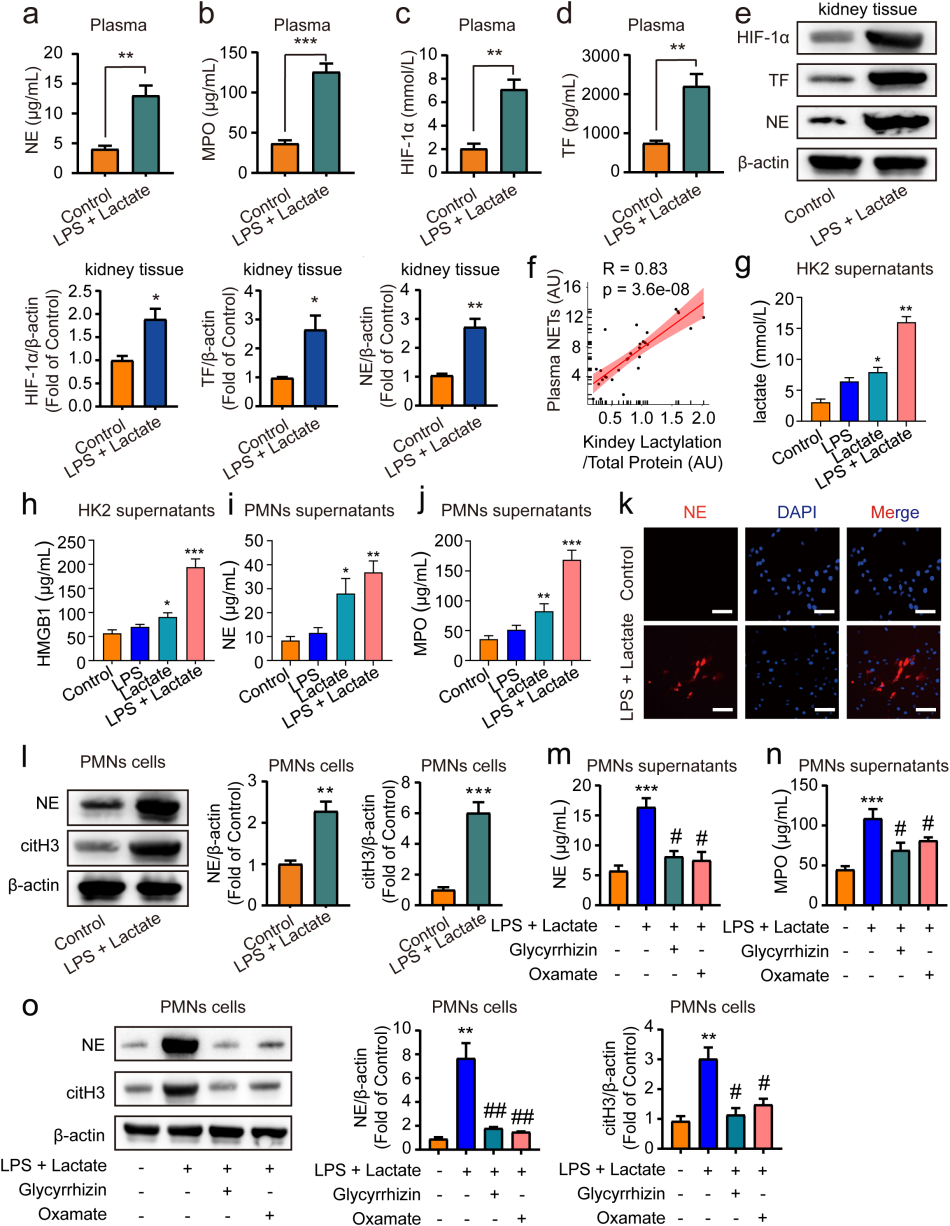
HMGB1 lactylation facilitates the release of NETs from neutrophil. ELISA kits were employed to measure NE levels **(A)**, MPO levels **(B)**, HIF-1α levels **(C)** and TF levels **(D)** in plasma. **(E)** Representative western blot bands and quantitative data demonstrating the expression of HIF-1α, TF and NE following 48 h treatment with lactate and low-dose LPS in kidney tissues in mice. **(F)** Pearson correlation analysis assessed the relationship between NETs levels and HMGB1 lactylation in mouse blood post-LPS + lactate treatment. **(G, H)** ELISA kits measured lactate and HMGB1 levels in HK2 cell supernatants. **(I, J)** ELISA kits quantitated NE and MPO levels in PMN cell supernatants. **(K)** Immunofluorescence data illustrate that conditioned media from HK2 cell supernatants incubated with PMN cells for 24 h significantly elevated NE expression levels in PMN cells. Scale Bar: 100 μm. Magnification: 200×. **(L)** Representative Western blot bands and quantitative data indicate that HK2 cell supernatants conditioned media significantly increased NE and citH3 expression levels in PMN cells after 24 h incubation. **(M, N)** ELISA results reveal that pre-treatment with glycyrrhizin or oxamate reversed the lactate and low-dose LPS induced increases in NE and MPO levels in PMN cell supernatants. **(O)** Representative Western blot bands and quantitative data showed that glycyrrhizin or oxamate could reverse the lactate and low-dose LPS-induced elevations of NE and citH3 in PMN cells. Significant difference was revealed following one-way ANOVA (^*^ *P* < 0.05, ^**^ *P* < 0.01 and ^***^ *P* < 0.001 vs. control group; ^#^ *P* < 0.05, ^##^ *P* < 0.01 vs. lactate + low-dose LPS treatment group; Bonferroni *post hoc* tests).

Previous research has demonstrated that HMGB1 can trigger the release of NETs from neutrophils (42), with excessive NETs formation further exacerbating AKI (43). Additionally, lactate has been shown to promote NETs production (44). Consequently, we sought to explore whether HMGB1 lactylation-mediated AKI in mice is associated with an enhanced generation of NETs. ELISA assays revealed that lactate treatment significantly increased levels of NE, MPO, HIF-1α, and TF in mouse plasma compared to control groups (**Figures 4A–D**). Additionally, levels of citH3 were also elevated in mouse plasma following treatment with lactate and low-dose LPS (**Supplementary Figure 1**). Moreover, Western blot analyses indicated that the expression of NE, HIF-1α, and TF were elevated following the combined application of lactate and low-dose LPS for 48 h in kidney tissue in mice (**Figure 4E**). Furthermore, Pearson correlation analysis indicated a positive correlation between NETs levels and HMGB1 lactylation following lactate treatment in mouse blood (**Figure 4F**). Further examination of the relationship between HMGB1 lactylation and NETs at the cellular level *in vitro* revealed that pre-treatment with lactate (50 μM) + low-dose LPS (10 nM) for 24 h significantly increased lactate and HMGB1 levels in supernatants of HK2 cells (**Figures 4G, H**). Additionally, lactate treatment alone for 24 hours also upregulated lactate and HMGB1 levels in HK2 cell supernatants *in vitro*, whereas this effect was not observed in the low-dose LPS alone group (**Figures 4G, H**). Moreover, conditioned medium from HK2 cells treated with lactate + low-dose LPS, when incubated with PMNs for 24 h, significantly boosted the expression levels of NE and MPO in PMNs (**Figures 4I, J**). This phenomenon was also present in the lactate alone treatment group; however, it was not found in the low-dose LPS alone group (**Figures 4I, J**). Immunofluorescence and immunoblotting analyses confirmed that NE and citH3 expression levels were elevated in PMNs following co-incubation with conditioned medium for 24 h (**Figures 4K, L**). Importantly, pre-treatment of HK2 cells with glycyrrhizin (50 μM) or LDH-A inhibitors (oxamate, 50 mmol/L) 2 h prior effectively abolished these effects (**Figures 4M–O**). Overall, these findings suggest that HMGB1 lactylation can trigger the release of NETs from neutrophils.

### 3.5 HMGB1 lactylation induces AKI via activation of the HMGB1-NETs signaling pathway

**Figure 5:**
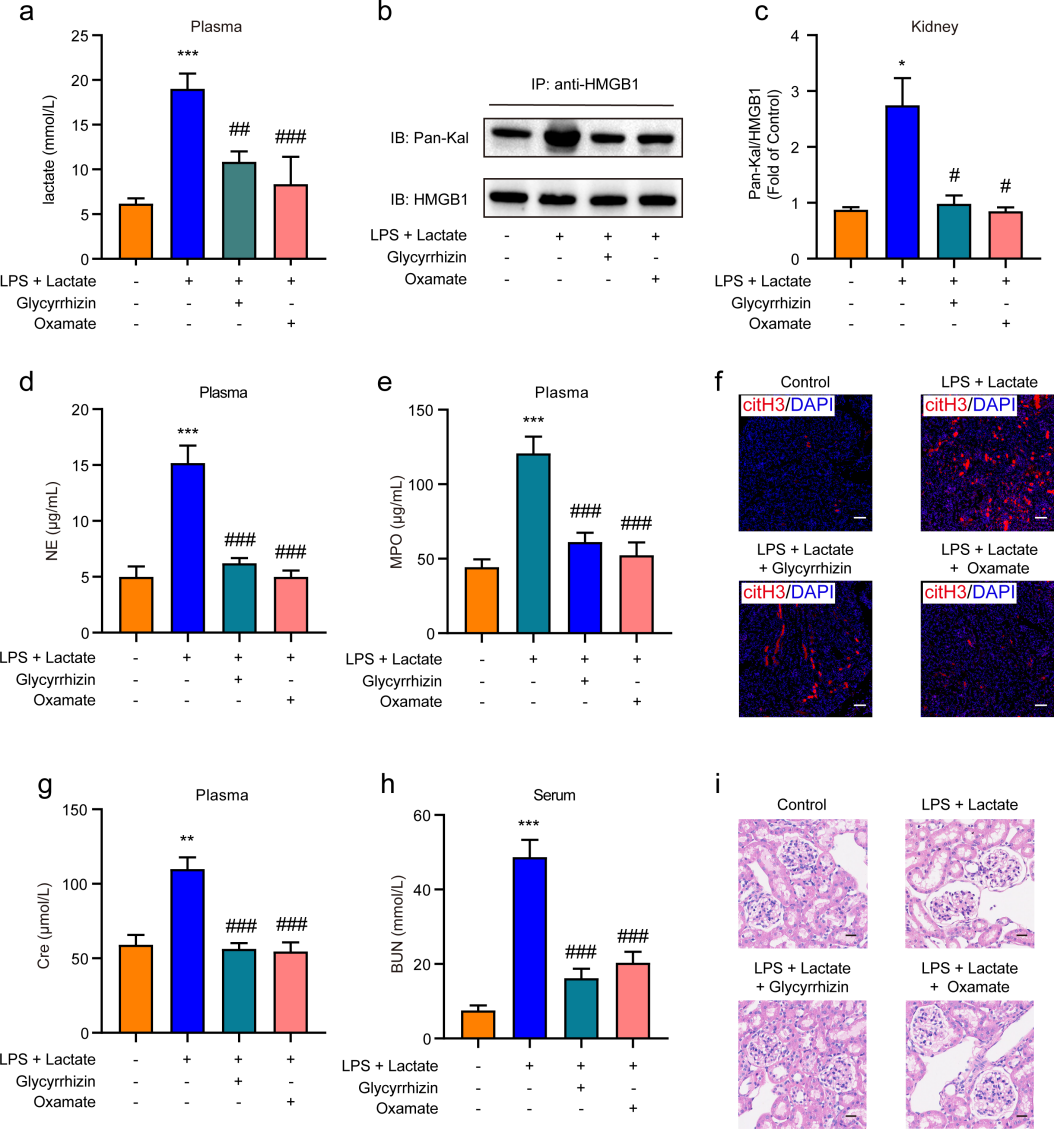
HMGB1 lactylation induces AKI by activating the HMGB1-NETs signaling pathway. **(A)** Plasma lactate levels were measured using ELISA kits. Mice received *i.p.* administration of glycyrrhizin (30 mg/kg) or oxamate (750 mg/kg) for 2 h, followed by combined treatment with lactate and low-dose lipopolysaccharide (LPS). **(B, C)** The effects of glycyrrhizin or oxamate on lactate and low-dose LPS induced HMGB1 lactylation in mice are shown. **(D, E)** ELISA kits were utilized to detect the levels of NE and MPO in plasma from mice. **(F)** Immunofluorescence analysis revealed the expression levels of citH3 in kidney tissues of mice. Mice received *i.p.* administration of glycyrrhizin (30 mg/kg) or oxamate (750 mg/kg) for 2 h, followed by combined treatment with lactate and low-dose LPS. **(G, H)** ELISA kits were employed to detect the levels of Cre and BUN in serum from mice. **(I)** HE stained sections of the kidney tissue from mice are depicted. Scale bar: 100 μm. Significant difference was revealed following one-way ANOVA (^*^ *P* < 0.05, ^**^ *P* < 0.01 and ^***^ *P* < 0.001 vs. control group; ^#^ *P* < 0.05, ^##^ *P* < 0.01 and ^###^ *P* < 0.001 vs. lactate + low-dose LPS treatment group; Bonferroni *post hoc* tests).

We further investigated whether HMGB1 lactylation induces AKI through the release of NETs in mice. *I.p.* administration of either HMGB1 inhibitor (glycyrrhizin, 30 mg/kg) or LDH-A inhibitor (oxamate, 750 mg/kg) was performed prior to the combined application of lactate and low-dose LPS to establish the AKI mouse model. The results indicated that pre-administration of either inhibitor significantly reduced plasma lactate levels (**Figure 5A**) and HMGB1 lactylation levels in kidney tissues (**Figures 5B, C**). In addition, both the HMGB1 and LDH-A inhibitors substantially inhibited the elevated levels of NE and MPO in mouse plasma induced by the combination of lactate and low-dose LPS (**Figures 5D, E**). Immunofluorescence results also confirmed that glycyrrhizin or oxamate could decrease the expression levels of citH3 in kidney tissues mediated by lactate and low-dose LPS (**Figure 5F**). Furthermore, both the HMGB1 and LDH-A inhibitors were found to reduce the expression levels of Cre and BUN in serum (**Figures 5G, H**). HE staining results further demonstrated that both inhibitors significantly mitigated the pathological alterations within kidney tissues provoked by lactate and low-dose LPS treatment, including considerable tubular injury characterized by cellular swelling and necrosis, prominent interstitial edema, and increased infiltration of inflammatory cells (**Figure 5H**). Collectively, these findings indicate that lactate accumulation-mediated HMGB1 lactylation in kidney tissue can induce AKI by activating the HMGB1-NETs signaling pathway.

## 4 Discussions

In this study, we identified lactate as an independent predictor of AKI in patients with ADHF by analyzing clinical and pathological data through the construction of a multivariate logistic regression model. This finding aligns with prior clinically relevant retrospective studies (45, 46). To deepen our understanding of these mechanisms, we also established a murine model of AKI using lactate. Notably, we observed that the combined administration of lactate and low-dose LPS led to a significant increase in plasma lactate levels, as well as elevated serum Cre and BUN levels. Moreover, proteomic analyses indicated that this combination augmented HMGB1 lactylation in renal tissue, an effect that could be reversed by administering HMGB1 siRNA. Additionally, HMGB1 lactylation was associated with an increased expression of NETs in the bloodstream. Pre-treatment with glycyrrhizin or oxamate effectively reversed the upregulation of NETs induced by lactate and low-dose LPS, as well as mitigated the lactate accumulation-mediated AKI. These findings suggest that lactate-induced HMGB1 lactylation may contribute to the pathogenesis of AKI in mice via the activation of the HMGB1-NETs signaling pathway.

HMGB1 is a position-dependent protein exhibiting varied functionalities based on its subcellular localization. Within the nucleus, HMGB1 serves as a DNA chaperone, ensuring the structural and functional integrity of chromosomes (47). In the cytoplasm, HMGB1 can enhance autophagy by interacting with the BECN1 protein (48, 49). Once released into the extracellular space, HMGB1 typically functions as a damage-associated molecular pattern (DAMP) molecule, modulating inflammation and immune responses through various receptors (50, 51). Under normal conditions, HMGB1 is predominantly localized in the nucleus, binding to chromatin; however, it can translocate to the cytoplasm and subsequently to the extracellular milieu during various stress scenarios (47). Following stimulation by factors such as LPS, HMGB1 may be actively secreted into the extracellular environment via vesicular transport methods (52). Additionally, HMGB1 can be passively released following various forms of cell death, including necrosis, ferroptosis, and apoptosis (47, 53). Extracellular HMGB1 can interact with TLR4 or RAGE, initiating signal transduction cascades that activate NF-κB, consequently facilitating the production of pro-inflammatory cytokines and contributing to various pathological conditions, including sterile inflammation (54), autoimmune disorders (55), septic shock (56), cancer (50), and AKI (57). Evidence indicates a positive correlation between serum HMGB1 levels and blood lactate concentrations, implying that lactate may regulate HMGB1 release (58). Furthermore, research has established that lactate functions as an epigenetic regulator, capable of adding a lactyl functional group to histones, thereby influencing specific gene expression profiles (30). HMGB1 has also been implicated in AKI progression (57, 59, 60). In our study, lactate was identified as an independent risk factor for AKI, corroborated by comprehensive clinical data analysis (**Figure 1**). Moreover, the combination of lactate and low-dose LPS markedly increased plasma lactate levels, elevated Cre and BUN in serum, and induced pathological changes within renal tissue (**Figure 2**). Mass spectrometry analysis demonstrated that lactate accumulation significantly raised HMGB1 lactylation levels in renal tissue, with HMGB1 gene knockdown leading to a reduction in lactylation levels and alleviation of AKI in mice (**Figure 3**).

The pathophysiology of AKI involves regulated cell death and inflammation (61). Notably, necroptosis, ferroptosis, and mitochondrial permeability transition-mediated regulated necrosis (MPT-RN) of tubular cells result in the release of DAMPs, leading to the recruitment of inflammatory cells and further injury (62). Among these inflammatory cells, neutrophils are notably prevalent during the early phases of ischemic AKI (63), and their depletion has been shown to prevent renal dysfunction, implicating their involvement in the progression of AKI (64). As key players in innate immunity, neutrophils combat pathogens through mechanisms including phagocytosis, degranulation, and cytokine release. Furthermore, neutrophils can trap and eliminate extracellular pathogens via the formation of NETs (65). NETs consist of a unique web of decondensed chromatin fibers infused with various antimicrobial factors (65). Initial studies suggested that the primary function of NETs is to capture and neutralize pathogens (65), however, emerging research has uncovered their potential to induce tissue damage (43, 66).

NETosis, the process of NETs formation, is energetically demanding, with glycolysis serving as a key energy source during this process (67). An increase in lactate accumulation has been correlated with enhanced NETs formation. Recent studies have further indicated that lactate facilitates the release of NETs from neutrophils (65, 68, 69), and the inhibition of lactate dehydrogenase has been shown to impede NETs generation (67, 69). Additionally, investigations have revealed a positive correlation between HMGB1 and lactate levels in the blood of critically ill patients experiencing septic infections (58). HMGB1 has also been shown to synergistically enhance NETs release in the presence of LPS (70). *In vitro* analyses involving cultured neutrophils demonstrated that recombinant HMGB1 can induce NETs formation (71). In coronary thrombosis, activated platelets has been observed to elevate HMGB1 production and incite NETs release from neutrophils (72). NETs have been identified as playing a pivotal role in AKI, with studies indicating that NETs can exacerbate kidney damage through the release of cytokines and histones (43). Notably, pre-treatment with NETs formation inhibitors has been shown to confer protection against ischemia-reperfusion injury (73, 74). we observed that lactate accumulation significantly elevated NETs levels in the plasma, with a positive correlation identified between NETs levels and HMGB1 lactylation. The combination of lactate and low-dose LPS also markedly increased lactate and HMGB1 levels in the supernatant of HK2 cells, and conditioned media collected from HK2 cell supernatants were able to induce NETs release in polymorphonuclear cells (PMNs) *in vitro*, effects that could be countered by HMGB1 or LDH-A inhibitors (**Figure 4**). Furthermore, *i.p.* injection of either HMGB1 inhibitor or LDH-A inhibitor led to reduced lactate levels, lowered HMGB1 lactylation in kidney tissue, and decreased serum levels of NETs, Cre, and BUN (**Figure 5**). These findings collectively illustrate that lactate accumulation-mediated HMGB1 lactylation is capable of inducing AKI in mice via the activation of the HMGB1-NETs signaling pathway.

In conclusion, our retrospective investigation substantiates lactate as an independent risk factor for AKI. Based on these findings, we conducted further experimental studies that demonstrated the capacity of lactate accumulation to induce AKI in murine models. The underlying mechanism potentially involves the activation of the HMGB1-NETs signaling cascade, which appears to be mediated by HMGB1 lactylation within renal tissues. A more comprehensive elucidation and characterization of these molecular pathways could prove instrumental in developing novel therapeutic strategies for ALI, given the established interconnection between renal and pulmonary dysfunction in critical illness.

## Data Availability

The data presented in the study are deposited in the iProX - integrated Proteome resources repository, accession number IPX0010716000.
