# Supplementary material for: HMGB1 lactylation drives neutrophil extracellular trap formation in lactate-induced acute kidney injury
Source: Front Immunol. 2025 Jan 9;15:1475543. doi: 10.3389/fimmu.2024.1475543 (PMC11754054; doi:10.3389/fimmu.2024.1475543)
Supplement: Supplementary file 1 [file Image1.pdf]

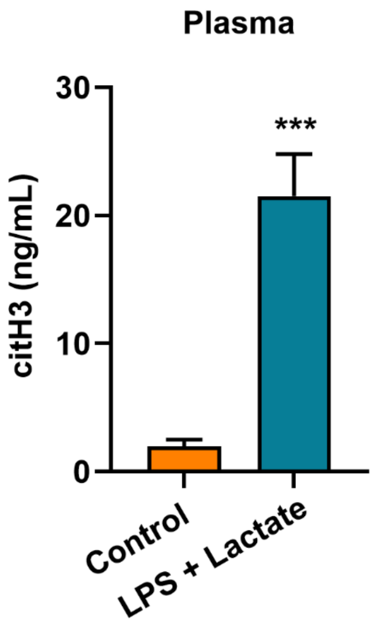

Supplementary Figure 1 ELISA kits were employed to measure citH3 levels in plasma. Significant difference was revealed following one-way ANOVA ($^{*}P < 0.05$ vs. control group; $^{\#}P < 0.05$; Bonferroni post hoc tests).
